# Impact of Surgeon Hand Dominance on Clinical and Functional Outcomes in Total Knee Arthroplasty: A Comparative Evaluation of Right and Left Knees

**DOI:** 10.7759/cureus.98459

**Published:** 2025-12-04

**Authors:** Ahmet Burak Satilmis, Tolgahan Cengiz

**Affiliations:** 1 Orthopaedics and Traumatology, Taşköprü State Hospital, Kastamonu, TUR; 2 Orthopaedics and Traumatology, Ordu Fatsa State Hospital, Ordu, TUR; 3 Orthopaedics and Traumatology, Ondokuz Mayıs University, Faculty of Medicine, Samsun, TUR

**Keywords:** arthroplasty, knee, osteoarthritis, surgeon handedness, treatment outcomes

## Abstract

Background: Total knee arthroplasty (TKA) is a commonly performed surgical intervention for end-stage gonarthrosis and aims to restore function, reduce pain, and improve quality of life. While the impact of surgical technique and implant selection on outcomes is well established, the potential role of surgeon ergonomics and hand dominance in influencing clinical outcomes has not yet been adequately investigated. This study compares the functional, clinical, and radiological outcomes of right and left TKA procedures performed under complete standard protocols by a right-handed surgeon who always operated from the right side of the surgical table.

Methods: This retrospective single-center study evaluated 100 patients (50 right, 50 left knees) who underwent primary TKA between 2020 and 2022 at the Department of Orthopedics and Traumatology, Kastamonu Taşköprü State Hospital, Kastamonu, Türkiye. Demographic, perioperative, functional, and radiological parameters were evaluated. Functional scores (Knee Injury and Osteoarthritis Outcome Score (KOOS), Western Ontario and McMaster Universities Arthritis Index (WOMAC), Kujala, Oxford), pain levels (Visual Analog Scale (VAS)), and range of motion (ROM) were recorded preoperatively, at one month and one year. Radiological results were evaluated using hip-knee-ankle angle (HKAA), medial distal femoral angle (MDFA), and medial proximal tibial angle (MPTA) measurements. Statistical comparisons were made using appropriate parametric and nonparametric tests.

Results: Both groups showed significant postoperative improvements in ROM, pain, and functional scores (p < 0.001). The right knee group had superior ROM at one year (p = 0.047), while the left knee group showed better KOOS and Oxford scores at early follow-up (p = 0.042 and p = 0.006, respectively). No significant difference was found in radiological alignment angles (p > 0.05), indicating consistent implant positioning. Hospital stay was longer in the left knee group (p = 0.011). These results show short- and long-term differences between right and left knee prostheses, but neither demonstrates overall clinical superiority.

Conclusion: Despite minor differences, the laterality of the operated knee did not significantly differ in overall outcomes. These findings emphasize that compliance with standard surgical technique, ergonomic consistency, and patient-specific planning are more effective in achieving TKA success than the surgeon's dominant hand.

## Introduction

Gonarthrosis, especially in the advanced form of osteoarthritis, is a chronic and destructive joint disease that dramatically reduces the quality of life of millions of people worldwide [1]. Pain, limited mobility, and loss of function radically impede patients' daily activities, leading to profound effects on social and economic life [2]. This condition causes patients to lose their ability to move independently and to suffer from the psychosocial impacts of mobility limitations in the long term. Therefore, the importance of effective surgical interventions that provide functional recovery is increasing.

Total knee arthroplasty (TKA) is an effective intervention that aims to reduce the effects of this destructive process and improve patients' quality of life. Shan et al. emphasized that correct surgical technique and implant placement strategies critically affect postoperative functional outcomes and implant life [3]; similarly, Sayah et al. reported that knee prosthesis applications significantly reduce patients' pain levels and increase functional capacity [4]. However, among the factors determining the success of knee prosthesis applications, perioperative management and rehabilitation strategies play an important role as much as surgical technique [5].

However, the success of knee prosthesis applications is closely related to the implant itself but also to the surgeon, surgical ergonomics, the technique applied, and meticulousness in patient selection. Meirhaghe et al. stated that the surgeon's hand dominance and the fixed positioning of the operating table positively affect the mechanical alignment of the implant and radiological results; Jaglarz et al. suggested that standardization of these elements could minimize the differences that may arise between right and left knees [6,7]. The surgeon's position during surgery has been identified as a potential variable affecting prosthesis placement accuracy and functional outcomes, especially for right-handed surgeons [8].

The success of TKA depends not only on implant design and surgical technique, but also on the surgeon's hand dominance and intraoperative ergonomics. It has been suggested that the surgeon's dominant hand and fixed position on the table may influence implant placement, functional recovery, and radiological outcomes. However, this effect has not been adequately investigated under standardized conditions. This study aims to demonstrate the potential effects of surgical ergonomics and hand dominance on clinical outcomes by comparing the results of right- and left-handed TKA performed by a right-handed surgeon who always operates from the right side of the table.

## Materials and methods

This single-center study is a retrospective examination conducted at the Department of Orthopedics and Traumatology, Kastamonu Taşköprü State Hospital, Kastamonu, Türkiye. TKA performed by the same surgeon, always positioned on the right side of the table, was evaluated considering right-hand dominance. A total of 100 patients with similar clinical and demographic characteristics between the groups were selected from 478 knee arthroplasty surgeries performed between 2021 and 2023. Patients were examined with separate data for both their right and left knees in light of clinical, radiological, and functional evaluations performed before and after surgery. The study is subject to local ethics committee approval, and signed informed consent was obtained from the participants. This study aimed to comprehensively evaluate whether the surgical side (right or left knee) in TKA influences clinical, functional, and radiological outcomes when operated on only the right side of the table by a single experienced, right-handed surgeon under standard conditions by comparing detailed perioperative parameters, patient-reported outcome measures, and radiographic alignment data in a homogeneously selected patient population.

Inclusion criteria included similar demographic characteristics, being 60 years or older, patients diagnosed with primary osteoarthritis who underwent TKA, having at least one year of follow-up data, and having the surgery performed by the same surgeon. Exclusion criteria included patients who had undergone revision surgery, had additional extremity injuries, or had systemic diseases that precluded application of standard rehabilitation protocols. There was no intergroup variation in the brand or model of the prosthetic implants utilized. Thus, the aim was to create a comparable data set for both right and left knee prostheses.

All surgeries were performed by the same experienced right-handed surgeon, who always approached the operating table from the right. Preoperative planning was performed according to standardized preoperative evaluation protocols and demographic characteristics; the American Society of Anesthesiologists (ASA) classification, body mass index (BMI), additional comorbidities, and radiological measurements of the patients were meticulously recorded. During the operation, the patients were positioned under spinal anesthesia, and all patients underwent knee replacement surgery using a standardized technique, which included the use of a tourniquet, a medial parapatellar approach, and the same implant brand. Perioperative data, including surgery duration, blood transfusion requirements, hospital stay, and early mobilization days, were collected in detail for both groups.

Data were collected from the patients at three time points: preoperative, one month postoperative, and one year postoperative. An independent orthopedist conducted functional and radiological evaluations. These evaluations included knee range of motion (ROM), Visual Analog Scale (VAS) pain score, Knee Injury and Osteoarthritis Outcome Score (KOOS), Western Ontario and McMaster University Osteoarthritis Index (WOMAC), Kujala anterior knee pain scale, and Oxford Knee Score [9]. Radiological evaluations were performed using angular measurements, including hip-knee-ankle angle (HKAA), medial distal femoral angle (MDFA), and medial proximal tibial angle (MPTA).

Ethics committee approval

The Local Ethics Committee approved this randomized controlled trial (date: 14 February 2025, decision no.: 2025/2126). The study was conducted in accordance with the Helsinki Declaration. Written informed consent has been obtained from the patients to publish this paper.

Statistical analysis

All statistical analyses were performed using IBM SPSS Statistics for Windows, version 24 (IBM Corp., Armonk, N.Y., USA) software. Categorical data are presented as frequency (n) and percentage (%), and continuous data are presented as mean, standard deviation, median, minimum, and maximum. Pearson's Chi-square and Fisher’s exact tests were used for group comparisons of categorical data. The conformity of continuous variables to the normal distribution was assessed with the Shapiro-Wilk test. An independent samples T-test was applied to compare the parametric independent variables between two groups. For variables that do not conform to a normal distribution, the Mann-Whitney U test was used for independent paired group comparisons, the Wilcoxon test was used for dependent paired group comparisons, and the Friedman test was used for dependent three-group comparisons. Correlation between continuous variables was assessed using Spearman correlation analysis. In all analyses, p < 0.05 was considered statistically significant.

## Results

The mean age of the 100 patients included in the study was 64.83 ± 4.30 years, and there was no significant difference in age between Group A, which underwent right knee prosthesis, and Group B, which underwent left knee prosthesis (p = 0.956). There was also no significant difference between the groups regarding essential demographic variables. such as BMI (body mass index), height, and weight (Table 1). When gender distribution was examined, 50% of the patients were male, and 50% were female, and a homogeneous distribution was observed between the groups regarding gender (p = 1.000).

**Table 1:** Comparison of clinical and demographic profiles between right and left total knee arthroplasty groups

Variable	Group A (right knee)	Group B (left knee)	Mann-Whitney U	Z	p-value	Effect size
	Mean ± SD	Mean ± SD				
Age (years)	64.82 ± 4.67	64.84 ± 3.93	1242	-0.05542	0.956	
Mean follow-up duration (months)	18.66 ± 5.96	16.44 ± 4.75	869	-265.596	0.008	0.27
Height (cm)	165.16 ± 9.30	166.18 ± 9.38	1190.5	-0.41126	0.681	
Weight (kg)	79.96 ± 10.93	79.48 ± 10.65	1218.5	-0.21754	0.828	
Operated knee circumference (cm)	57.84 ± 5.05	56.46 ± 4.76	1078	-118.935	0.234	
Non-operated knee circumference (cm)	56.9 ± 4.3	56.3 ± 4.82	1147	-0.71304	0.476	
Total hospital stay (days)	3.32 ± 1.15	4 ± 1.5	913.5	-25.476	0.011	0.25
Operation time (minutes)	61.48 ± 4.26	62.36 ± 4.43	1084.5	-114.647	0.252	

When evaluated in terms of chronic disease, the presence of additional disease, diabetes (DM), and goiter frequency was not different between the two groups (p = 1.000, p = 0.311, and p = 0.577, respectively). At the same time, hypertension (HT) was more common in patients who underwent left knee prostheses (62.3%, p = 0.016). Asthma was also more prevalent in patients who underwent left knee prostheses, but this difference was insignificant (p = 0.092) (Table 2). Regarding the ASA score, most patients were in the ASA 2 category (91%), and no significant difference was found between the groups (p = 1.000). When the hospitalization period was evaluated, the hospitalization period of patients who underwent left knee prosthesis was found to be significantly longer compared to the right knee group (p = 0.011). No difference was found between the two groups regarding blood transfusion requirement (p = 0.386), and most patients (87%) did not need transfusion.

**Table 2:** Comparison of patients' comorbidities between the right and left knee arthroplasty groups

Variable	Category	Group A (right knee)	Group B (left knee)	Total
Gender	Male	25 (50.0%)	25 (50.0%)	50 (50.0%)
	Female	25 (50.0%)	25 (50.0%)	50 (50.0%)
ASA score	I	5 (10.0%)	4 (8.0%)	9 (9.0%)
	II	45 (90.0%)	46 (92.0%)	91 (91.0%)
Comorbidity	(-)	17 (34.0%)	16 (32.0%)	33 (33.0%)
	(+)	33 (66.0%)	34 (68.0%)	67 (67.0%)
Diabetes mellitus	(-)	26 (52.0%)	32 (64.0%)	58 (58.0%)
	(+)	24 (48.0%)	18 (36.0%)	42 (42.0%)
Hypertension	(-)	30 (60.0%)	17 (34.0%)	47 (47.0%)
	(+)	20 (40.0%)	33 (66.0%)	53 (53.0%)
Hypothyroidism	(-)	41 (82.0%)	44 (88.0%)	85 (85.0%)
	(+)	9 (18.0%)	6 (12.0%)	15 (15.0%)
Asthma	(-)	48 (96.0%)	42 (84.0%)	90 (90.0%)
	(+)	2 (4.0%)	8 (16.0%)	10 (10.0%)

Knee joint ROM was evaluated in three periods: preoperative, first month, and first year. ROM scores increased significantly in both groups (p < 0.001). However, it was determined that the ROM scores of patients who underwent right knee prosthesis were higher than those of patients who underwent left knee prosthesis as of the first year (p = 0.047). This suggests that patients who underwent right knee prosthesis may have a better ROM in the long term. VAS scores measuring pain levels were examined in preoperative, first month, and first year. VAS scores decreased significantly in both groups (p < 0.001). Still, as of the first year, the VAS scores of patients who underwent right knee prosthesis were considerably higher than those who underwent left knee prosthesis (p = 0.049). This finding suggests that patients who underwent right knee prosthesis may feel more pain in the long term. There was no statistically significant difference between the right (Group A) and left (Group B) knee arthroplasty groups in terms of HKAA, MDFA, and MPTA angles (p > 0.05). This result shows that mechanical alignment was achieved similarly after surgery in both groups, and there was no side-dependent difference in implant placement (Table 3).

KOOS and Oxford scores in terms of functional assessment decreased significantly in the postoperative period (p < 0.001). It was determined that patients who underwent left knee prostheses had higher values, especially in terms of first-month KOOS scores (p = 0.042). Similarly, Oxford scores also showed a significant difference in favor of patients who underwent left knee prostheses in the first month (p = 0.050) and the first year (p = 0.006). These findings suggest that patients who underwent left knee replacement showed better functional recovery in the short term. By contrast, although WOMAC and Kujala scores improved over time, no significant difference was found between the groups (p > 0.05) (Table 3).

When the effect of gender on the recovery process was examined, it was determined that female patients experienced more significant pain reduction (VAS score, p = 0.002) and functional recovery (KOOS score, p = 0.001) compared to male patients as of the first month. However, no significant difference was found in terms of changes in VAS and KOOS scores between patients who underwent right and left knee replacement (p > 0.05). This suggests that gender may be effective in the recovery process after knee replacement.

When the factors related to the operation time were evaluated, a negative correlation was found between age and operation time (r = -0.265, p = 0.008), meaning that the operation time was longer in younger patients. In addition, a moderate positive correlation was found between height and weight and operation time, and it was determined that the operation time increased as height and weight increased (r = 0.463 for height, r = 0.432 for weight, p < 0.001). However, there was no difference in operation time between the right and left knee prosthesis groups (p = 0.242)

There are some clinical and functional differences between Group A (right knee prosthesis) and Group B (left knee prosthesis), but no group showed a clear superiority in general. Patients who underwent right knee prostheses had a higher ROM in the long term (first year) (p = 0.047). By contrast, patients who underwent left knee prostheses showed better functional recovery in the short term (first month) and had higher KOOS and Oxford scores (p = 0.042 and p = 0.006). When evaluated in terms of pain level, patients who underwent right knee prosthesis had higher VAS scores in the first year, meaning they could feel more pain in the long term (p = 0.049). The duration of hospitalization was significantly longer in patients who underwent left knee prostheses (p = 0.011), and hypertension was also more common in this group (p=0.016). In terms of gender, it was observed that female patients experienced more significant pain reduction and functional recovery compared to males. These results show differences in the short- and long-term effects of right and left knee prosthesis applications. Still, there is no significant difference in superiority regarding general clinical outcomes.

**Table 3:** Comparative analysis of functional, radiological, and pain-related outcomes between right and left total knee arthroplasty groups ROM: range of motion, VAS: Visual Analog Scale, HKAA: hip-knee-ankle angle, MDFA: medial distal femoral angle, MPTA: medial proximal tibial angle, KOOS: Knee Injury and Osteoarthritis Outcome Score, WOMAC: Western Ontario and McMaster Universities Osteoarthritis Index, Preop: preoperative, Postop: postoperative

Variable	Group A (right knee)	Group B (left knee)	Mann-Whitney U	Z	p-value	Effect size
	Mean ± SD	Mean ± SD				
Knee ROM (Preop)	89.6 ± 13.51	86.9 ± 18.12	1168.5	-0.59394	0.553	
Knee ROM (1st month postop)	116.2 ± 7.8	113.6 ± 9.42	1066.5	-137.819	0.168	
Knee ROM (1st year postop)	120 ± 7.56	115.9 ± 9.67	981	-198.214	0.047	0.20
VAS Score (Preop)	9.44 ± 0.97	9.26 ± 1.26	1197.5	-0.42714	0.669	
VAS Score (1st month)	3.7 ± 2.3	3.68 ± 1.94	1235.5	-0.10197	0.919	
VAS Score (1st year)	1.42 ± 0.99	1.02 ± 0.94	976.5	-196.959	0.049	0.20
HKAA (°)	178.86 ± 2.73	179.62 ± 2.63	1034	-149.793	0.134	
MDFA (°)	87.51 ± 1.39	87.49 ± 1.55	1237	-0.08962	0.929	
MPTA (°)	86.90 ± 3.36	87.01 ± 3.62	1225	-0.17235	0.863	
KOOS (Preop)	102.28 ± 30.13	106.28 ± 30.33	1153.5	-0.66575	0.506	
KOOS (1st month)	39.86 ± 19.22	46.6 ± 20.27	956	-202.865	0.042	0.20
KOOS (1st year)	23.86 ± 16.19	27.42 ± 15.51	1036.5	-147.386	0.141	
WOMAC (Preop)	52.78 ± 21.56	50.44 ± 19.43	1161	-0.61463	0.539	
WOMAC (1st month)	14.44 ± 12.24	17.1 ± 12.64	1083	-115.365	0.249	
WOMAC (1st year)	8.08 ± 10.85	8.58 ± 8.42	1062	-130.916	0.190	
Kujala score (Preop)	39.98 ± 14.93	38.46 ± 14.43	1110.5	-0.96302	0.336	
Kujala (1st month)	66.5 ± 9.6	65.46 ± 10.34	1169	-0.56014	0.575	
Kujala (1st year)	72.84 ± 8.77	67.68 ± 19.93	1027	-154.305	0.123	
Oxford score (Preop)	36.64 ± 11.99	39.52 ± 10.76	1080	-117.407	0.240	
Oxford (1st month)	19.9 ± 5.84	22.76 ± 7.6	967	-195.984	0.050	0.20
Oxford (1st year)	16.74 ± 5.83	19.46 ± 5.69	859	-274.404	0.006	0.27

## Discussion

TKA is critical in pain management and improving the quality of life in treating advanced osteoarthritis. Neuprez et al. emphasized the impact of surgical technique and implant compatibility on postoperative quality of life; this study was prepared with similar principles [10]. In our study, the results of right and left knee arthroplasty performed by the same surgeon from the right side of the table, always considering right-hand dominance, were examined in detail. Tanzer et al. stated that preoperative planning is the basis of success; we pay attention to patient selection and plan accordingly [11]. Preoperative patient demographics, BMI, ASA classification, and meticulous recording of additional diseases increased the methodological soundness of our study. Magone et al. stated that patient selection directly affects the operation results and demonstrated the importance of this approach [12]. In addition, the patient range in our study shows a homogeneous distribution in terms of age, BMI, and other demographic characteristics. This situation further increased the reliability of the comparison between the two knees. The literature has reported that surgical technique and implant compatibility play a critical role in the success of TKA [3,4], and that the surgeon's hand dominance and table position can also affect mechanical alignment [6,7,13,14]. However, the impact of these factors on clinical and functional outcomes has been limitedly investigated. Our study filled this gap by comparing the results of right and left knee TKA under standard conditions and demonstrated that ergonomic differences are not decisive.

The effect of the surgeon's dominant hand on the results of TKA is a topic of debate in the literature. In a study conducted by Saiki et al., the femoral and tibial implantation accuracy of different navigation systems was compared, and it was stated that the surgeon's hand dominance could lead to potential asymmetries in implant placement [13]. In addition, Kim et al. reported higher postoperative pain in the second knee surgery in staged bilateral TKA applications. These findings indicate that the surgeon's hand dominance and position may impact surgical outcomes, especially in bilateral TKA [14]. In our study, although there were some significant differences between the short- and long-term clinical results of right and left knee prostheses applied in a fixed position by a right-handed surgeon, differences that were not decisive in overall results were observed.

Using a fixed right side of the table during surgery revealed the surgeon's ergonomic advantage. Jaglarz et al. reported that the surgeon's ergonomic position affected the quality of implant placement; in this context, the application of the standard protocol was aimed [6]. Perioperative parameters such as operation time, need for blood transfusion, and hospital stay did not show a statistically significant difference between the two groups. Compagnoni et al. emphasized that meticulous perioperative management reduces complication rates and supports recovery [15]. In surgeries we performed per standard surgical techniques and protocols, the effect of variation in surgical position on the operation results could be minimized. Also, Jaglarz et al. stated that differences in surgical position may lead to unexpected variations in the operative results [6]; in our study, on the contrary, no significant difference was observed between the groups.

In the postoperative period, the patient's pain and functional improvements were evaluated using VAS, KOOS, WOMAC, Kujala, and Oxford knee scores. Rupp et al. stated that these scores are objective indicators of surgical success; we also observed similar improvement trends in both knees [9]. Regarding functional improvement, Shan et al. reported that pain reduction and increased range of motion significantly improved patients' quality of life after knee replacement [3]. Our study significantly improved the right and left knees compared to the preoperative period. We believe that early rehabilitation is also essential for the functional success of the patients in our study. Early mobilization and systematic rehabilitation programs are critical in functional recovery after knee arthroplasty. Jiao et al. reported that early rehabilitation reduces complication rates and accelerates recovery [16].

We performed radiologic evaluations, especially with HKAA, MDFA, and MPTA measurements. Dhungana et al. revealed the importance of correct mechanical alignment on implant life and clinical outcomes [17], while our data also showed consistent results between the two knees. Dhungana et al. emphasized the role of mechanical alignment in reducing postoperative complications [17]; in this study, no significant difference was observed between the right and left knees in radiological angle measurements. Some studies have suggested that the surgeon's right-hand dominance and positioning on the right side of the table may lead to potential asymmetric results while indicating that ergonomic factors may affect implant placement, showing that standard protocols can minimize this effect [18]. As a result of the data we obtained in our study, we did not detect any radiological differences between the groups.

Multicenter randomized studies provide more comprehensive data on surgeon ergonomics and patient distribution; Foster et al. have proven that implant technologies and compatible protocols can achieve tremendous success in this area [19]. In addition, the data obtained show that improvements in the right and left knees parallel the manufacturing processes in the literature. Haffar et al. stated that standard protocols that minimize the effects of surgical ergonomics can reduce the difference between the two knees [20]. This study shows no significant difference between the results of right and left knee prostheses under standard protocols performed by a right-handed surgeon from the right side of the table. These findings emphasize the importance of meticulously implementing surgical techniques, perioperative management, and rehabilitation programs.

The greatest strength of this study is that the results of knee prostheses performed by the same surgeon based on right-hand dominance with a fixed surgical protocol were meticulously compared between the right and left knees. This situation shows how effective standardization of surgical technique, ergonomics, and perioperative management can achieve equal clinical and functional results between the two sides. Advantages include carefully planning the study methodology, ensuring a homogeneous distribution of patient demographics and clinical parameters, and implementing standard rehabilitation protocols with meticulous preoperative evaluation. In this way, the potential effects of the surgeon's right-hand dominance and positioning on the right side of the table could be examined by isolating them from the impact of other factors. On the other hand, limitations include the single-center and retrospective nature of the study, the limited sample size, and the lack of long-term follow-up data for a larger population. In addition, the fact that the single surgical technique applied by the surgeon was not compared with the methods used by different surgeons or different centers creates limitations in the generalizability of the results obtained. Therefore, future multicenter prospective studies will increase the applicability of study findings to larger patient populations.

## Conclusions

Despite theoretical concerns about surgical ergonomics and hand dominance, this study reveals that when TKA is performed under meticulously standardized conditions by a right-hand dominant surgeon, the operated side-right or left-does not significantly alter clinical, radiological, or functional outcomes. Although minor variations were observed, such as better early functional scores in left knees and slightly superior long-term range of motion in right knees, no consistent or clinically meaningful superiority was established. These findings underscore that the true determinant of success in knee arthroplasty lies not in the laterality of the procedure but in the precision of technique, the consistency of perioperative management, and the surgeon’s adherence to standardized protocols.
